# Supplementary material for: spatzie: an R package for identifying significant transcription factor motif co-enrichment from enhancer–promoter interactions
Source: Nucleic Acids Res. 2022 Jan 31;50(9):e52. doi: 10.1093/nar/gkac036 (PMC9122533; doi:10.1093/nar/gkac036)
Supplement: gkac036_Supplemental_File [file gkac036_supplemental_file.pdf]

Supplementary Material

spatzie: An R package for identifying significant transcription factor motif co-enrichment from enhancer-promoter interactions

Jennifer Hammelman[1,2,*], Konstantin Krismer[2,3,*] and David K. Gifford[2,3,4,†]

[1]Computational and Systems Biology, Massachusetts Institute of Technology, 77 Massachusetts Avenue, Cambridge, MA 02139, USA
[2]Computer Science and Artificial Intelligence Laboratory, Massachusetts Institute of Technology, 32 Vassar Street, Cambridge, MA 02139, USA
[3]Department of Biological Engineering, Massachusetts Institute of Technology, 77 Massachusetts Avenue, Cambridge, MA 02139, USA
[4]Department of Electrical Engineering and Computer Science, Massachusetts Institute of Technology, 77 Massachusetts Avenue, Cambridge, MA 02139, USA

* These authors contributed equally to this work.
† To whom correspondence should be addressed. Tel: +1 617 253 6039; Email: gifford@mit.edu

1 Supplementary Figures

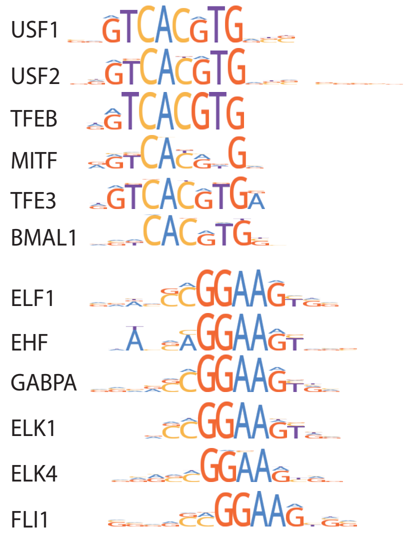

Supplementary Figure S1: Transcription factor motif position weight matrices that are discovered to have significant co-enrichment from USF1 and ELF1 simulated enhancer:promoter interaction task show high motif similarity to USF1 and ELF1 motifs.

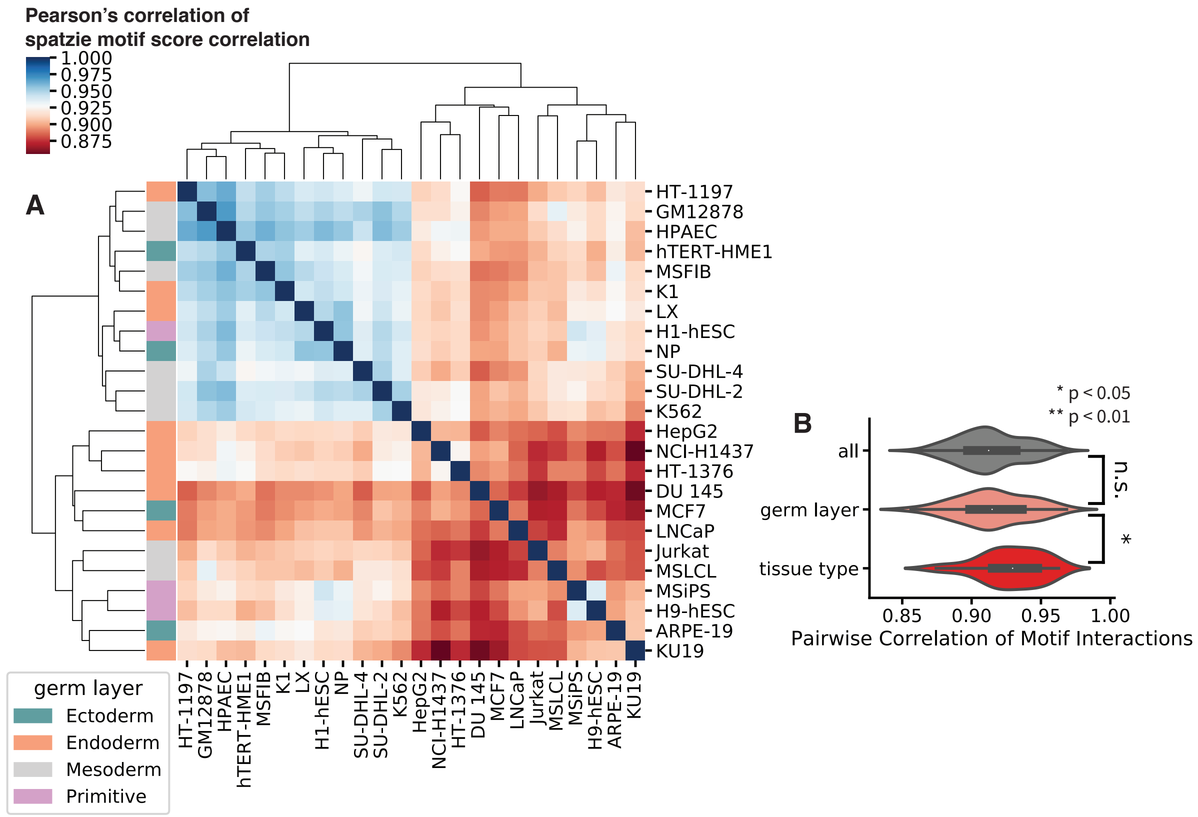

Supplementary Figure S2: **(A)** Pearson correlation of spatzie motif score of CID discovered enhancer:promoter interactions for RAD21 ChIA-PET of 24 human cell types. **(B)** Pairwise correlation of spatzie co-enriched motifs from CID enhancer:promoter interactions are higher among samples that are from the same tissue.

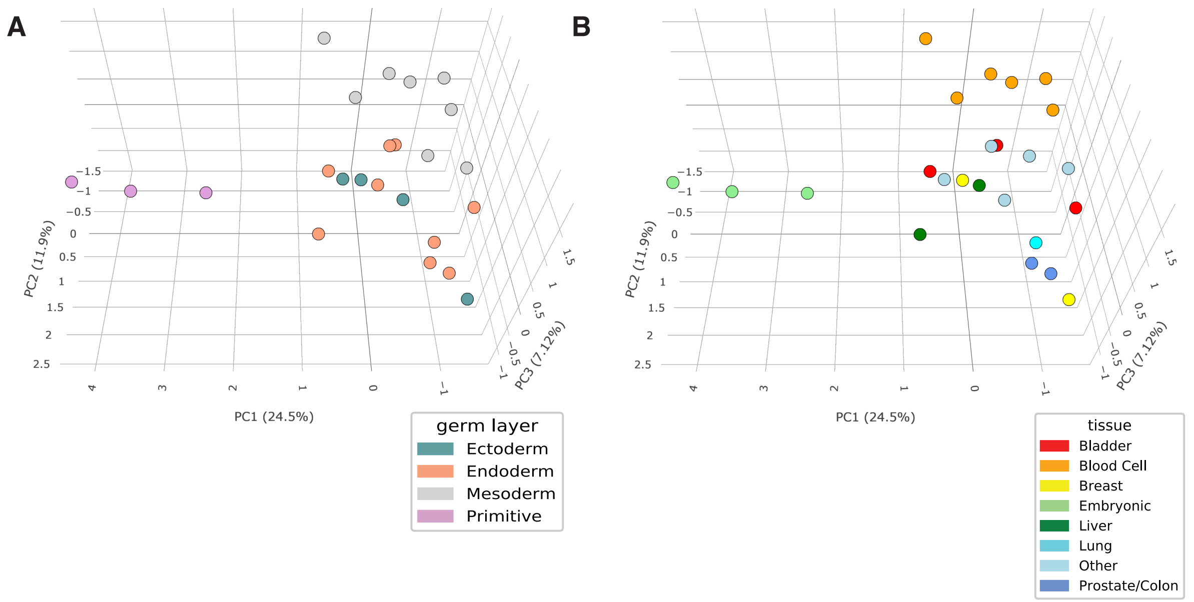

Supplementary Figure S3: PCA on spatzie discovered transcription factor interactions shows **(A)** germ layer and **(B)** tissue type clustering. Plotted are the first three principal components with 25.5 %, 11.9 %, and 7.12 % explained variance.

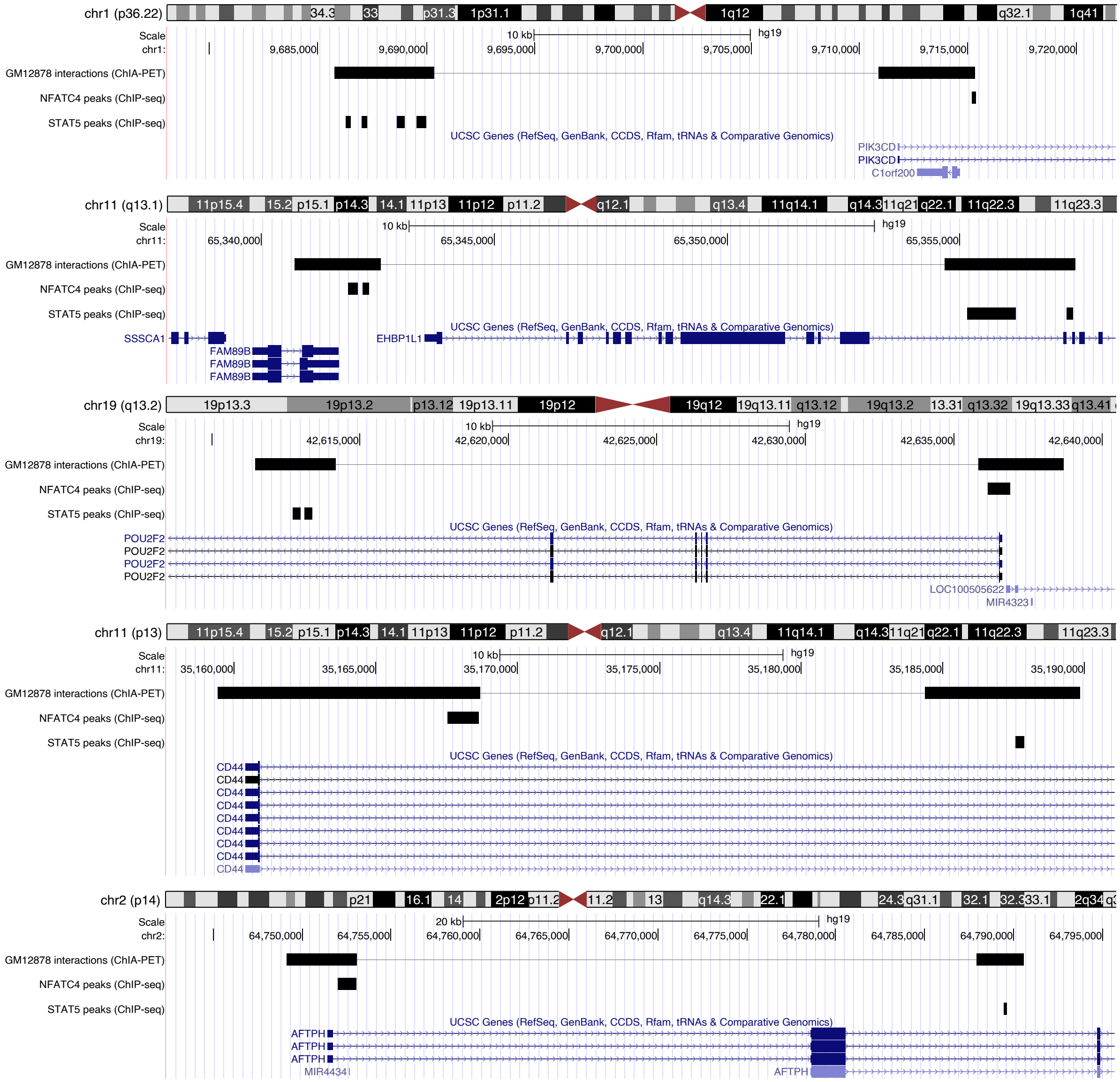

Supplementary Figure S4: Examples of motif co-occurrence of gene-proximal/promoter NFATC4 peaks and gene-distal/enhancer STAT5 peaks that are located in the respective anchors of reproducible genomic interactions obtained by ChIA-PET experiments in GM12878 cells.

2 Supplementary Tables

dataset label	Target	Cell line	GEO/ENCODE identifier
USF1 rep1	ChIP	MEL	ENCFF996MWJ
USF1 rep2	ChIP	MEL	ENCFF550MZG
ELF1 rep1	ChIP	MEL	ENCFF186NAS
ELF1 rep2	ChIP	MEL	ENCFF592ERV
ARPE	RAD21	ARPE-19	GSE134745
DU145	RAD21	DU-145	GSE134745
MSFIB	RAD21	fibroblast	GSE134745
GM12878	RAD21	GM12878	GSE134745
H1	RAD21	H1	GSE134745
H9	RAD21	H9	GSE134745
LX	RAD21	hepatocyte	GSE134745
HepG2	RAD21	HepG2	GSE134745
HT1197	RAD21	HT-1197	GSE134745
HT1376	RAD21	HT-1376	GSE134745
HMTERT	RAD21	hTERT-HME1	GSE134745
Jurkat	RAD21	Jurkat-Clone-E6-1	GSE134745
AKTHY	RAD21	K1	GSE134745
K562	RAD21	K562	GSE134745
KU19	RAD21	KU-19-19	GSE134745
LNCAP	RAD21	LNCAP	GSE134745
MCF7	RAD21	MCF-7	GSE134745
MSIPS	RAD21	MSiPS	GSE134745
MSLCL	RAD21	MSLCL	GSE134745
H1437	RAD21	NCI-H1437	GSE134745
NP	RAD21	neural progenitor cells	GSE134745
ECS	RAD21	pulmonary artery endothelial cells	GSE134745
DHL2	RAD21	SU-DHL-2	GSE134745
DHL4	RAD21	SU-DHL-4	GSE134745

Supplementary Table S1: ChIP-seq and ChIA-PET datasets used in this study.

cell type	interactions		reproducible interactions
	replicate 1	replicate 2	
HT-1197	87,937	68,978	24,184 (30.8 %)
SU-DHL-2	78,277	62,513	17,272 (24.5 %)
GM12878	86,457	76,072	18,876 (23.2 %)
SU-DHL-4	75,226	51,953	13,834 (21.8 %)
HT-1376	55,916	59,273	9642 (16.7 %)
NP	76,689	75,488	11,577 (15.2 %)
K562	61,333	60,257	8605 (14.2 %)
hTERT-HME1	60,590	65,466	8774 (13.9 %)
LX	71,679	71,043	9653 (13.5 %)
NCI-H1437	54,742	58,519	6985 (12.3 %)
KU19	51,392	63,260	5810 (10.1 %)
MSFIB	74,630	73,401	6896 (9.3 %)
ARPE-19	73,702	42,810	4721 (8.1 %)
MSiPS	51,602	40,523	2422 (5.3 %)
K1	62,580	84,760	3704 (5.0 %)
MSLCL	74,774	42,038	2174 (3.7 %)
HepG2	51,051	45,550	1704 (3.5 %)
H1-hESC	50,622	88,466	2268 (3.3 %)
HPAEC	90,604	57,452	1826 (2.5 %)
LNCaP	47,873	50,221	1190 (2.4 %)
Jurkat	43,199	51,338	1142 (2.4 %)
MCF7	45,457	50,532	1153 (2.4 %)
DU 145	39,122	60,616	1024 (2.1 %)
H9-hESC	49,896	51,245	837 (1.7 %)

Supplementary Table S2: Number of identified and reproducible interactions per ChIA-PET experiment. Reproducible interactions are interactions present in both replicates and with an IDR < 0.05. Reproducible interactions were identified with IDR2D [3].

3 Supplementary Methods

For analysis of human RAD21 ChIA-PET data for co-enrichment of motifs underlying enhancer:promoter interactions, we applied spatzie to both the interactions that were provided by Grubert et al. using a custom method for generating a unified interaction set from many ChIA-PET samples and then using PET support to call cell type-specific interaction events as well as a unified interaction set that was generated by calling interactions with CID [1].

3.1 Calling interaction events with CID

For each ChIA-PET experiment, we first used Mango 1.2.1 [4] (downloaded from `https://github.com/dphansti/mango`) to remove linker sequences and reads potentially due to polymerase chain reaction duplication, and aligned the raw reads using bowtie 1.2.3 (steps 1 - 3 in Mango pipeline). Mango was executed with the *reportallpairs* flag and the recommended parameter settings for the ChIA-PET Tn5 tagmentation protocol: *-keepempty TRUE -maxlength 1000 -shortreads FALSE*. CID (downloaded from `https://groups.csail.mit.edu/cgs/gem/versions.html`) was then run on the BEDPE file produced by Mango, using default parameters. Lastly, we used MICC [2] to assess the significance of all interactions identified by CID that are supported by more than one PET read.

3.2 Generating a unified CID interaction set

For the CID interaction set, we first ran CID independently on all 48 samples (24 cell types; 2 replicates for each cell type). We then took the union of interactions called in all 48 samples. To merge overlapping interactions, we independently merged overlapping anchor1 and anchor2. We then removed interactions containing anchors that were larger than 20kb in size or interactions where as an artifact of merging, anchor1 and anchor2 overlapped. This resulted in a set of 71,643 joint CID interactions.

3.3 Calling CID enhancer:promoter interactions for each cell type

We then obtained PET support from each of the 48 samples for the set of joint CID interactions and based on the correlation between replicates removed two samples (H1 rep2 and pulomonary artery endothelial cell rep2) that did not appear to have consistent correlation with their other cell type replicate and cell types within the same tissue. To obtain the interactions for each cell type from our joint CID interactions set, we looked at PET support and replicate correlation. For samples with two replicates, we called an interaction event within that cell type if it had greater than 1 PET in both replicates. For samples with one replicate, (H1 and pulomonary artery endothelial cell), we called an interaction event if the MICC FDR value of the event was less than 0.05. Interaction events were then filtered to those where one anchor was within 2.5kb of a promoter and the other was promoter distal (not within 2.5kb of a promoter). The selection criteria for calling cell type-specific allowed us to obtain a sufficient number of enhancer:promoter interaction events to run spatzie for co-motif enrichment (range 2,178-14,113 enhancer:promoter interactions per cell type) and were consistent with the range in the number of enhancer:promoter events discovered in Grubert et al. (range 1,476-11,850 enhancer:promoter interactions per cell type).

3.4 Analysis of spatzie results for CID and Grubert et al. interactions

After co-enrichment of motifs at enhancer:promoter interactions was computed with spatzie, we analyzed the correlation between co-enrichment scores. For Grubert et al. interactions, we used only motif pairs where co-enrichment was significant under multiple hypothesis correction in at least one cell type. For CID interactions, we used all motif pairs as limiting to interactions that were significant under multiple hypothesis correction resulted in less meaningful clustering of motif co-enrichment scores by tissue type.

References

[1] Y. Guo, K. Krismer, M. Closser, H. Wichterle, and D. K. Gifford. High resolution discovery of chromatin interactions. *Nucleic Acids Res*, 47(6):e35, 04 2019.

[2] C. He, M. Q. Zhang, and X. Wang. MICC: an R package for identifying chromatin interactions from ChIA-PET data. *Bioinformatics*, 31(23):3832–3834, Dec 2015.

[3] K. Krismer, Y. Guo, and D. K. Gifford. IDR2D identifies reproducible genomic interactions. *Nucleic Acids Res*, 48(6):e31, 04 2020.

[4] D. H. Phanstiel, A. P. Boyle, N. Heidari, and M. P. Snyder. Mango: a bias-correcting ChIA-PET analysis pipeline. *Bioinformatics*, 31(19):3092–3098, Oct 2015.
